# Cell-based artificial platelet production: historical milestones, emerging trends, and future directions

**DOI:** 10.1007/s44313-025-00071-9

**Published:** 2025-05-27

**Authors:** Kyoung Mi Kim, Koudai I. Albaira, Jayoung Kang, Yong Gon Cho, Soon Sung Kwon, Jaecheol Lee, Dae-Hyun Ko, Sinyoung Kim, Seung Yeob Lee

**Affiliations:** 1 Department of Laboratory Medicine, Jeonbuk National University Medical School and Hospital, Jeonju, Korea; 2 Research Institute of Clinical Medicine of Jeonbuk National University, Biomedical Research Institute of Jeonbuk National University Hospital, Jeonju, Korea; 3 Department of Laboratory Medicine, Yonsei University College of Medicine, Seoul, Korea; 4 School of Pharmacy, Sungkyunkwan University, Suwon, Korea; 5 Biomedical Institute for Convergence at SKKU (BICS), Sungkyunkwan University, Suwon, Korea; 6 Department of Laboratory Medicine, Asan Medical Center, University of Ulsan College of Medicine, Seoul, Korea

**Keywords:** Cell-based artificial platelets, Human pluripotent stem cells, Hematopoietic stem cells, Megakaryocytes

## Abstract

Cell-based artificial platelet production has made remarkable progress over the past three decades, driven by the need for safe and stable platelet sources in the face of donor limitations and transfusion-related risks. This review provides a chronological overview of the evolution of in vitro platelet production from various cell sources (CD34+ hematopoietic stem cells, embryonic stem cells, induced pluripotent stem cells (iPSCs), and others) and highlights key advances in the field. We outline developments from the foundational experiments of the 1990s, through the introduction of iPSCs in the mid-2000s, to the adoption of three-dimensional culture and bioreactor technologies in the late 2010s and the emergence of clinical trials in the 2020s. In addition, we discuss future perspectives, including the role of advanced gene editing and scalable biomanufacturing technologies in accelerating clinical translation. This comprehensive review underscores the promise of artificial platelet production technologies for clinical applications and discusses the remaining challenges, such as scalability, cost-effectiveness, and regulatory hurdles. The recent completion of the first human clinical trials using iPSC-derived platelets marks a significant milestone, pointing to a future in which patient-specific or human leukocyte antigen-universal platelets may be transformed into transfusion medicine and regenerative therapies.

## Introduction

Cell-based artificial platelet production research has received increasing attention in hematology, regenerative medicine, and bioengineering. Platelets, derived from megakaryocytes in the bone marrow, play a crucial role in hemostasis and preventing excessive bleeding [1]. Thrombocytopenia, characterized by a critically low platelet count, can result from various etiologies, such as autoimmune disorders, drug reactions, bone marrow failure syndromes, or viral infections. Platelet transfusion is commonly used to treat or prevent complications in patients with thrombocytopenia [2]. However, the short 5-day shelf life of platelets and the risks of immune reactions or infections pose significant challenges, necessitating the development of new methods for a stable platelet supply [3, 4].

To achieve efficient in vitro platelet production, researchers have explored the optimization of culture conditions (mimicking in vivo niches), the administration of differentiation-inducing growth factors, and the evaluation of platelet functionality and morphology [5, 6]. Furthermore, breakthroughs in gene-editing technologies have bolstered the clinical applicability of artificially produced platelets by enhancing production yields and potentially reducing transplant rejection through customized or human leukocyte antigen (HLA)-universal platelets [7]. This review synthesizes the latest research on megakaryocyte differentiation and platelet production from various cellular sources, including CD34+ hematopoietic stem cells (HSCs), human embryonic stem cells (hESCs), and human induced pluripotent stem cells (iPSCs) [8]. The revolutionary advent of iPSCs, which can reprogram mature somatic cells into a near-embryonic state, has unlocked new possibilities for personalized medicine and markedly impacted the development of novel platelet therapies [9, 10]. Finally, we outline emerging perspectives on how continuing innovation may drive more efficient, scalable, and safe artificial platelet therapies.

By exploring these progressive technological developments (Table 1), this review provides key insights into how emerging methods promise to address platelet supply challenges, reduce infectious and immunological risks, and improve patient outcomes. Ongoing innovations in artificial platelet production will remain at the forefront of hematology and regenerative medicine with the prospect of transforming the standard of care in platelet transfusion and beyond.

**Table 1:** Chronological Overview of Cell-Based Artificial Platelet Production

Decade	Cell Source	Representative Cytokines and Growth Factors	Products	Representative Product Yields	Product Testing Methods	Significance	References
1990–1999	CD34+ HSC	Serum Meg-CSA, IL-3, IL-6, IL-9, MGDF (TPO)	MK, PLT	4 MK/CD34+ HSC to 2 × 10^6 MK 3.2 × 10^6 platelet-sized fragments/mL < 50 PLT/MK	**Morphological characterization:** Romanowsky staining, EM, immunofluorescence **Polyploidy:** PI, DAPI **MK/PLT markers:** CD34, CD41, CD42b, CD61 **Proplatelet detection:** EM **PLT count:** hemocytometer, flow cytometry **Viability:** trypan blue **Aggregation:** microscopy **Activation:** P-selectin, PAC-1	Established in vitro MK production and initial PLT generation from MK; advanced serum-free cultures; demonstrated cord blood CD34+ cells as a robust source for MK	[11–15]
2000–2009	CD34+ HSC, ESC, MK cell line, adipocyte precursor cells	TPO, SCF, Flt3-L, IL-6, IL-11, IL-1β, bFGF, SDF-1, PDGF, FGF-4	MK, PLT	5–20 × 10^3 MK to 2 × 10^6 MK 15 × 10^4 PLT to 1.68 × 10^11 PLT	**Morphological characterization:** Romanowsky staining, rhodamine-phalloidin, EM **Polyploidy:** PI, DAPI **MK/PLT markers:** CD34, CD41, CD42a, CD42b, CD61 **Proplatelet detection:** HMC **HLA expression analysis:** flow cytometry **Viability:** calcein-AM **Aggregation:** LTA **Activation:** P-selectin, PAC-1 **Tumorigenicity:** CFU assay	Methods to generate MK/PLT using various cell sources under different conditions (ESC, fetal liver–derived CD34+, adipocyte precursors); introduced large-scale ex vivo strategies, temperature adjustments, and key signaling pathways (e.g., c-MYC, VEGF) for enhanced yield	[16–25]
2010–2019	iPSC, CD34+ HSC, ESC, fibroblast, ASCL	TPO, IL-3, IL-6, IL-7, IL-9, IL-10, IL-11, SCF, VEGF, Flt3-L, bFGF, BMP-4	MKP, MK, ProPLT, PLT	100–800 to 138,331 ± 61,031 MKP > 16 MKP/iPSC to 2.5 × 10^10 MK 1,907.67 ± 196.90 ProPLT 6 PLT/MK to 4.2 (±0.5) × 10^7 PLT	**Morphological characterization:** Romanowsky and Hoechst staining, EM **Polyploidy:** PI, DAPI, FISH **MK/PLT Markers:** CD9, CD31, CD34, CD41, CD42a, CD42b, CD63 **Platelet count**: flow cytometry **Viability:** calcein-AM, 7-AAD **β2 m and HLA class I expression:** flow cytometry **Aggregation:** LTA **Activation:** P-selectin, PAC-1, GPVI **Phenotype analysis:** megakaryocyte colony forming assay **In vivo tests:** transfusion assay, intravital imaging, teratoma testing	HLA class I–deficient platelets from CD34+ progenitors; large-scale hESC-derived platelets; direct fibroblast reprogramming into MK-like cells; varied methods (pH shifts, shear stress in bioreactors, 3D rotary culture) to increase yield; transcription-factor cocktails (GATA1, FLI1, TAL1) to boost MK/PLT generation; established new potential cell sources (fibroblasts, adipose stem cells)	[26–46]
2020–present	iPSC, CD34+ HSC	TPO, SCF, IL-3, IL-6, IL-11, GM-CSF, bFGF, VEGF, activin A, BMP-4, Flt3-L	MK, PLT	2.5 × 10^4 MK to 2–4 × 10^7 MK 3 iPLAT yield/MK to 15 PLT/MK	**Morphological characterization:** EM, immunocytochemistry **Polyploidy:** PI **MK/PLT Markers:** CD31, CD41, CD42b, CD61 **β2 m and HLA class I expression:** flow cytometry **Aggregation:** flow cytometry, LTA **Activation:** P-selectin, PAC-1, Annexin V **Clot formation:** ROTEM **Immunogenicity test:** NK cell co-culture assays **In vivo tests:** transfusion assay, bleeding assay	CRISPR/Cas9-driven β2m knockout for HLA class I–deficient iPSC-platelets; roller-bottle expansion for large-scale MK production; streamlined hiPSC-derived platelet yield boosted by activin A, BMP-4, CHIR, and VEGF; EHMT inhibitors to enhance MEP and MK specification; phase 1 clinical trials of iPSC-platelets show safety and feasibility in humans	[47–51]

*Abbreviations*: *HSC* hematopoietic stem cell, *MK* megakaryocyte, *PLT* platelet, *ESC* embryonic stem cell, *Flt3-L* Flt3-ligand, *iPSC* induced pluripotent stem cell, *ASCL* human adipose-derived mesenchymal stromal/stem cell line, *MKP* megakaryocyte progenitor, *CFU* colony-forming unit, *EM* electron microscopy, *HMC* Hoffman modulation contrast microscopy, *LTA* light transmission aggregometry, *EHMT* euchromatic histone lysine methyltransferase, *MEP* megakaryocyte-erythroid progenitor

## Evolving trends and statistics in cell line utilization

**Fig. 1:**
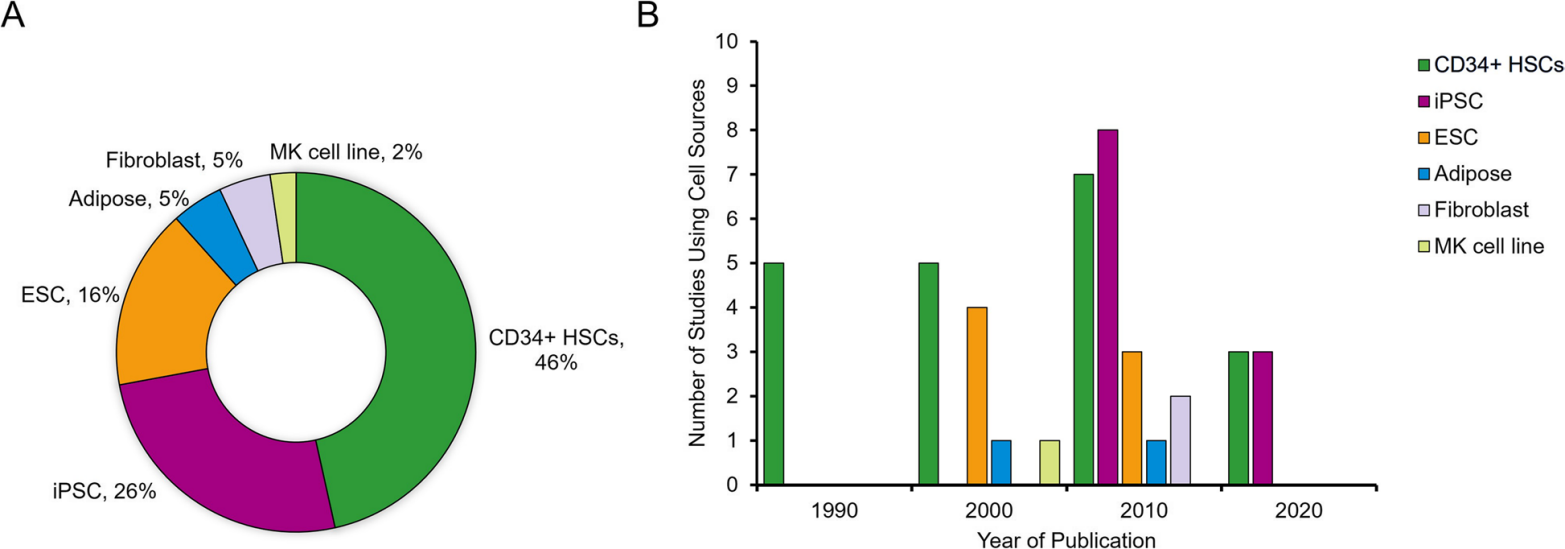
Changes in cell line statistics and usage trends. (A) Distribution of cell sources used across 41 studies from 1990s to 2020s. Of these, 46% used CD34+ hematopoietic stem cells (HSCs), 26% used induced pluripotent stem cells (iPSCs), 16% used embryonic stem cells (ESCs), and 5% each used adipose-derived cells and fibroblast cells. (B) Timeline illustrating how these cell sources were adopted in different periods. CD34+ HSCs have been widely used since the 1990s, while iPSCs rose to prominence in the 2010s following their initial development in 2006. See Table 1 for the full list of the 41 included studies.

Megakaryocytes arise from a hierarchical hematopoietic process, wherein multipotent HSCs first give rise to megakaryocyte-erythroid progenitors (MEPs), which then mature into megakaryocytes capable of producing platelets. In artificial platelet research, megakaryocytes are derived from multiple cell sources through the coordinated action of growth factors, cytokines, and genetic manipulations. The efficiency and yield may differ significantly when producing platelets from these sources. Among the 41 relevant studies published between 1990 and 2023, 46% used CD34+ HSCs, 26% used iPSCs, 16% used ESCs, and 5% each used adipose-derived and fibroblast cells (Fig. 1A; See Table 1 for the full list of the 41 included studies). Looking at the timeline, CD34+ HSCs (being more readily accessible from peripheral blood, bone marrow, and umbilical cord blood) have been commonly used since the 1990s [11–18]. In the early 2000s, ESCs and adipose-derived cells were added to the list of cell sources (Fig. 1B). ESCs, derived from the inner cell mass of blastocysts, can differentiate into various cell types, including megakaryocytes, under defined culture conditions [19–22]. Similarly, adipose-derived cells, isolated from adult adipose tissue, can be induced to differentiate into megakaryocytes through treatment with specific growth factors and cytokines [41]. The advent of iPSC technology in 2006 revolutionized this field, and iPSCs became a major focus area in the 2010s [26, 27]. A key reason for this shift involved growing ethical concerns regarding deriving ESCs from blastocysts and the desire to move away from potentially immunogenic allogeneic sources. In contrast, iPSCs can be generated from a patient’s own somatic cells, bypassing many ethical controversies, while also offering personalized and immune-compatible platelet products. In addition to iPSCs, HSCs have remained a frequent research subject, leading to substantial advancements. By the 2020s, CD34+ HSCs and iPSCs remained the dominant primary cell sources for platelet production research.

## Early stages and basic research (1990s)

The 1990s marked the inception of cell-based artificial platelet research, mainly centered on defining methodologies for deriving megakaryocytes from peripheral blood CD34+ HSCs [11, 12]. Investigations focused on understanding normal platelet production and replicating these processes in vitro, including the use of human serum in culture media. Differentiation was typically evaluated by examining the expression of glycoprotein (GP) IIb/IIIa or GP Ib/IX/V or the presence of polyploid cells. The effects of various growth factors and cytokines (interleukin (IL)−3, IL-6, stem cell factor (SCF), and thrombopoietin (TPO)) on megakaryocyte and platelet production were tested, while serum-free conditions were also explored [13, 14]. Additional studies highlighted that megakaryocyte proliferation can be stimulated by cultivating CD34+ cells isolated from the peripheral blood, bone marrow, or cord blood. For instance, CD34+ cells treated with megakaryocyte growth and development factors in serum-free culture produced a high ratio of megakaryocyte precursors in just 14 days [15]. These foundational experiments laid the groundwork for subsequent innovations in platelet production.

## Expansion of HSC-based research (2000s to 2010s)

From the early 2000s, research broadened to include megakaryocyte differentiation and platelet production using CD34+ HSCs, embryonic stem cells (ESCs), and adipose-derived precursors. HSCs harvested from peripheral blood, umbilical cord blood, and bone marrow were frequently used. For example, Ma et al. (2000) investigated how TPO influences the proliferation and maturation of megakaryocytes sourced from the fetal liver and adult bone marrow, focusing on the involvement of cyclins in megakaryocyte endoreplication [23]. In 2006, Matsunaga et al. proposed a three-step method that produced 210–350 times more platelets than classical ex vivo approaches when using CD34+ cells from cord blood, demonstrating that these in vitro–generated platelets were morphologically and functionally similar to peripheral blood platelets [16]. Studies also assessed how different cytokine combinations (e.g., TPO + IL-6 + IL-1 beta (IL-1β) + SCF) significantly improved platelet yields [17], while culture temperature modulations (e.g., 39°C) boosted platelet output by more than 16-fold compared with standard 37°C cultures [18]. These findings underscored the importance of precisely controlled culture conditions and growth factor regimens for optimizing platelet production.

Concurrently, methods for producing megakaryocytes and platelets from ESCs began to emerge. Eto et al. (2002) demonstrated for the first time that a large number of megakaryocytes can be generated from murine ESCs using an OP9 stromal co-culture system supplemented with TPO, IL-6, and IL-11 [19]. These ESC-derived megakaryocytes exhibited hallmark features such as polyploidy, proplatelet formation, and the expression of key platelet-specific markers including integrin αIIbβ3 and GPIbα. Fujimoto et al. (2003) also produced platelets from mouse ESCs, identifying primitive and definitive megakaryocytic lineages [20]. Subsequent studies cultured hESCs for megakaryocyte and platelet production under the influence of TPO [21, 22]. Gaur et al. generated 5–20 × 10^3 megakaryocytes per 1 × 10^5 hESCs, while Takayama et al. produced up to 4.8 (±0.2) × 10^6 platelets. To dissect the roles of integrin subunits in megakaryocyte biology and platelet function, researchers utilized lentiviral transduction to stably overexpress genes of interest (e.g., αIIbβ3), facilitating the exploration of inside-out and outside-in signaling pathways [17, 21]. This approach allows the precise control of integrin expression levels, enabling functional assays that measure fibrinogen binding, proplatelet formation, and downstream signaling events. Consequently, lentiviral overexpression of integrin subunits has significantly broadened our understanding of how megakaryocytes mature and produce fully functional platelets at the molecular level.

Researchers also developed new ex vivo culture systems that utilize various cell sources. One approach involved culturing UT-7/TPO megakaryocytic cells and CD34+ CD38^lo cells in the presence of TPO and SU6656 to produce functional platelet-like fragments; however, these fragments lacked the typical architecture of native platelets under electron microscopy [24]. Another study cultivated adipocyte precursor cells from subcutaneous fat tissue and CD34+ cells from bone marrow, resulting in approximately 2 × 10^6 megakaryocytes and 15 × 10^4 platelets from 10^7 adipose precursor cells [25]. However, of note, the absolute numbers of platelets reported vary widely owing to differences in quality control, cell counting methods, and culture conditions among laboratories; therefore, direct comparisons between studies should be interpreted with caution. Nevertheless, these advances significantly refined the culture conditions, enhancing the quantity and quality of in vitro–derived platelets.

## Emergence of iPSCs (Early 2010s)

The early 2010s witnessed the consolidation of iPSC technology as a revolutionary catalyst for artificial platelet research. Although CD34+ HSCs remain the mainstay, iPSCs, ESCs, fibroblasts, and adipose cells became prominent sources of megakaryocytes and platelets. A hallmark study in 2010 demonstrated that hematopoietic progenitor cells can be manipulated to produce functional HLA class I–deficient platelets [28], highlighting the potential immune-evasive benefits of downregulating these molecules, which normally present endogenous peptides to cytotoxic T lymphocytes. This breakthrough opened a promising avenue for avoiding alloimmune complications.

Takayama et al. (2010) refined protocols for iPSC-derived platelet (iPLAT) production by genetically modifying iPSC lines (e.g., OCT3/4-Kusabira Orange, SOX2-EGFP, KLF4-EGFP, and c-MYC) [26]. Their work highlighted that transient but timely activation and subsequent downregulation of c-MYC could bolster platelet yields, indicating a pivotal role of c-MYC in driving megakaryocyte differentiation from iPSCs. In 2014, Nakamura et al. expanded this concept by using c-MYC, BMI1, and BCL-XL to generate proliferative megakaryocyte precursors (imMKCLs), successfully releasing functional platelets [27]. These studies demonstrated the feasibility of large-scale patient-specific platelet production, potentially eliminating the risks of donor-related immune rejection and infection.

The use of ESCs for platelet production also matured. Lu et al. (2011) showed that combined IL-6, IL-9, IL-11, basic fibroblast growth factor (bFGF), vascular endothelial growth factor (VEGF), TPO, SCF, and bone morphogenetic protein-4 (BMP-4) could stimulate hESC-derived megakaryocyte production, yielding approximately 6.7 ± 0.4 platelets per megakaryocyte [29]. However, direct comparisons should be approached with caution because the absolute numbers of platelets generated depend on the different classification systems and quality control measures used in each study. Further attempts at serum-free and feeder-free protocols advanced clinical translation efforts [30]. To enhance yields, Nakagawa et al. (2013) developed bioreactor approaches that applied dual-flow shear stress, substantially increasing platelet production relative to standard two-dimensional cultures [31]. Collectively, these breakthroughs in iPSC and ESC research laid a robust foundation for ongoing attempts to balance the yield, safety, and clinical feasibility of artificial platelets.

## Development of HLA-universal megakaryocytes (Mid-2010s)

Building on the 2010 demonstration of HLA class I–deficient platelets, mid-2010s research placed a stronger emphasis on HLA-universal megakaryocytes. Feng et al. (2014) successfully generated cryopreservable megakaryocyte precursors and platelets with null expression of major HLA class I molecules (A, B, and C) under serum- and animal component–free conditions [32]. Similar work by Borger et al. (2016) used shRNA targeting β2-microglobulin (β2m) to produce iPSC-derived megakaryocytes with significantly reduced expression of HLA class I molecules [33]. These genetically modified megakaryocytes retained polyploid levels comparable to those of non-manipulated controls, although they were still lower than those of fully mature megakaryocytes in vivo. They also displayed the ability to form proplatelets and showed strong resistance to complement- or cell-dependent cytotoxicity.

Simultaneously, new three-dimensional (3D) culture systems demonstrated that culturing CD34+ cells in a 3D environment fosters better megakaryocyte maturation and platelet yield [34]. Researchers also explored direct cellular differentiation strategies; for example, Moreau used GATA1, FLI1, and TAL1 to boost megakaryocyte generation from human pluripotent stem cells, and Pulecio et al. (2016) employed core transcription factors (Gata2, Runx1, Gata1, Tal-1, Lmo2, and c-Myc) to convert fibroblasts directly into megakaryocyte-like cells [35, 36]. These advances not only expanded the range of cell sources available for platelet production but also provided alternative approaches for generating megakaryocyte-like cells with potentially distinct functional properties, offering more streamlined and personalized manufacturing options.

## Application of 3D culture and bioreactor technology (Late 2010s)

From the late 2010s, researchers increasingly used 3D culture methods and bioreactor platforms to accelerate large-scale platelet production. Yang et al. (2016) studied a rotating cell culture system that significantly enhanced megakaryopoiesis and thrombopoiesis of umbilical cord blood CD34+ cells, which was approximately 3.7-fold more efficient than static culture [37]. Blin et al. (2016) designed a microfluidic device with von Willebrand factor-coated micropillars to capture megakaryocytes and subject them to controlled shear forces, producing approximately 3.7 platelet-like particles per megakaryocyte at a throughput of over one million megakaryocytes per chip [38]. To standardize and scale megakaryocyte differentiation, Perdomo et al. (2017) developed a serum-free protocol that led to the production of > 90% pure megakaryocytes within approximately 10–12 days, generating between 19 and 42 platelets per CD34+ cell input [39]. Although these yields are promising, the numbers reported (e.g., fold increases and platelets per cell) are based on laboratory-specific quality control criteria and counting methodologies, which may vary across studies.

Hansen et al. (2018) reported a reproducible feeder-free monolayer system capable of producing high-purity megakaryocytes (and other hematopoietic lineages) from single-cell–derived iPSC colonies [40]. Around the same time, Tozawa et al. (2019) used adipose-derived stem cells to generate functional megakaryocytes through endogenous TPO secretion and activation of critical transcription factor pathways [41]. Collectively, these studies integrated refined differentiation protocols with cutting-edge bioreactors and culture devices, moving closer to therapeutically meaningful platelet yields for clinical use.

## Clinical application of platelet therapeutics (2020s)

By the 2020s, scientists had significantly advanced the efficiency and purity of megakaryocyte differentiation and platelet production, thereby yielding an expanded range of potential clinical applications. Building on earlier HLA-KO iPSC-platelet initiatives, Suzuki et al. (2020) enhanced their clinical potential by generating iPLATs designed to avoid natural killer (NK) cell activation and circulate comparably to wild-type platelets in humanized mice [47]. These iPLATs generated approximately three platelets per megakaryocyte, underscoring continuous improvements in genetic manipulation and scaling methods.

Around the same time, Norbnop et al. (2020) utilized CRISPR/Cas9 to disrupt β2m, creating iPSC-derived HLA class I–universal platelets detectable in mice up to 24 h post-transfusion [48]. Other groups investigated innovative culture platforms to enrich megakaryocyte yields [49] or developed protocols to differentiate and harvest approximately 338 platelets per iPSC [50]. Parallel studies on CD34+ cells also demonstrated functional in vitro–generated platelets that recirculate and assist in hemostasis [52]. Investigations into epigenetic regulators, such as EHMT inhibitors, further multiplied megakaryocyte and platelet numbers by six- to eight-fold [53], although the absolute platelet and megakaryocyte numbers generated should be explained in terms of the standards used.

Recent efforts have aimed to develop personalized platelets for patients with unique alloimmune refractoriness, such as those with rare HPA-1 mismatches [54]. Using the imMKCL expansion platform, researchers have performed non-clinical testing under Good Manufacturing Practice (GMP) conditions, including pathogen, tumorigenicity, and toxicity assessments, followed by a phase 1 clinical trial of autologous iPLATs [55]. These trials confirmed safety after a one-year follow-up, with no adverse events, and detected larger platelets (iPLATs) in the peripheral blood by flow cytometry, although no increase in the corrected count increment was observed after transfusion [56]. Inspired by these results, allogeneic iPLATs clinical trials are currently underway, signifying a transformative era in platelet transfusion therapy.

## Future perspectives

Although significant advances have been made in the production of functional platelets from various sources, ongoing innovations are crucial for their widespread clinical adoption. Next-generation genetic editing methods, such as base and prime editing, could enhance the creation of HLA-universal megakaryocytes while minimizing off-target effects [32, 33]. Further refinements in 3D bioreactor design, microfluidic platforms, and automated culture systems are required to achieve higher yields and reduce manufacturing complexity [57, 58].

In addition, the development of cost-effective reagents, standardized protocols, and robust quality control measures is essential to ensure scalability and compliance with regulatory standards, including GMP guidelines for cellular therapies [56]. GMP compliance requires rigorous facility requirements, validated production processes, and comprehensive documentation to ensure product consistency and patient safety. Similarly, manufacturing constraints, such as limited bioreactor capacity and high production costs, continue to present challenges that must be addressed for clinical translation [35, 54, 55]. Moreover, commercial-scale production can be hindered by the complexity of supply chains for growth factors and expenses associated with maintaining aseptic conditions, highlighting the need for innovative cost-lowering strategies [42].

Future studies should also consider regulatory and ethical issues, including obtaining informed consent for the use of patient-derived cells and navigating the patent landscape surrounding starting cell types and gene-editing technologies employed in platelet generation in vitro [9]. As personalized medicine gains momentum, integrating patient-specific iPSC lines with robust immunomodulatory strategies could pave the way for custom-tailored platelet therapies, ultimately transforming transfusion medicine and improving patient outcomes globally [26].

## Conclusion

This review presents a comprehensive account of human cell–based artificial platelet research, tracing its history from the early exploration of peripheral blood progenitor cells to contemporary innovations involving iPSC and 3D bioreactor technologies. Steady progress in differentiation protocols, genetic engineering, and culture systems has significantly improved the yield, function, and clinical applicability of platelets. Furthermore, as highlighted in our future perspectives, advanced gene-editing techniques and scalable culture platforms could propel these technologies toward broader clinical adoption.

These developments hold immense promise for addressing the persistent challenges in platelet supply, transfusion-related infections, and immunological complications. The refinement of iPSC-driven methods has opened new avenues for personalized therapy, particularly through HLA-universal platelets that minimize graft rejection. Although issues such as manufacturing costs, immune modulation, and regulatory complexity persist, ongoing technological innovations and encouraging clinical trial data indicate a bright future for artificial platelet products. As this field continues to advance, cell-based platelet production has become the mainstay of precision medicine, improving the quality of life and survival of numerous patients worldwide.

## Data Availability

No datasets were generated or analysed during the current study.
